# Effectiveness of midwifery-led care on pregnancy outcomes in low- and middle-income countries: a systematic review and meta-analysis

**DOI:** 10.1186/s12884-023-05664-9

**Published:** 2023-05-26

**Authors:** Rekiku Fikre, Jessica Gubbels, Wondwosen Teklesilasie, Sanne Gerards

**Affiliations:** 1 Department of Health Promotion, Faculty of Health, Medicine & Life Science, Maastricht University, NUTRIM School of Nutrition and Translational Research in Metabolism, Maastricht, Netherlands; 2 School of Public Health, Hawassa University College of Medicine and Health Science, Hawassa, P.Box:1560 Ethiopia

**Keywords:** Midwifery, Pregnancy, Childbirth, Meta-analysis, Systematic review, Low- and Middle-income countries

## Abstract

**Background:**

Midwifery-led care is an evidence-based practice in which a qualified midwife provides comprehensive care for low-risk pregnant women and new-borns throughout pregnancy, birth, and the postnatal period. Evidence indicates that midwifery-led care has positive impacts on various outcomes, which include preventing preterm births, reducing the need for interventions, and improving clinical outcomes. This is, however, mainly based on studies from high-income countries. Therefore, this systematic review and meta-analysis aimed to assess the effectiveness of midwifery-led care on pregnancy outcomes in low- and middle-income countries.

**Methods:**

We used the Preferred Reporting Items for Systematic Review and Meta-Analysis (PRISMA) guidelines. Three electronic databases (PubMed, CINAHL, and EMBASE) were searched. The search results were systematically screened by two independent researchers. Two authors independently extracted all relevant data using a structured data extraction format. Data analysis for the meta-analysis was done using STATA Version 16 software. A weighted inverse variance random-effects model was used to estimate the effectiveness of midwifery-led care on pregnancy outcomes. Odds ratio with a 95% confidence interval (CI) was presented using a forest plot.

**Results:**

Ten studies were eligible for inclusion in this systematic review, of which five studies were eligible for inclusion in the meta-analysis. Women receiving midwifery-led care had a significantly lower rate of postpartum haemorrhage and a reduced rate of birth asphyxia. The meta-analysis further showed a significantly reduced risk of emergency Caesarean section (OR = 0.49; 95% CI: 0.27–0.72), increased odds of vaginal birth (OR = 1.14; 95% CI: 1.04–1.23), decreased use of episiotomy (OR = 0.46; 95% CI: 0.10–0.82), and decreased average neonatal admission time in neonatal intensive care unit (OR = 0.59; 95% CI: 0.44–0.75).

**Conclusions:**

This systematic review indicated that midwifery-led care has a significant positive impact on improving various maternal and neonatal outcomes in low- and middle-income countries. We therefore advise widespread implementation of midwifery-led care in low- and middle-income countries.

**Supplementary Information:**

The online version contains supplementary material available at 10.1186/s12884-023-05664-9.

## Background

Pregnant women should have access to high-quality maternal health care, which is a fundamental human right [1]. However, every day, nearly 830 women die worldwide from preventable causes related to pregnancy and childbirth. The majority of deaths occur in low- and middle-income countries, accountable for 95% of maternal deaths and 90% of all children’s deaths worldwide [2, 3].

The expansion of basic and comprehensive emergency obstetric care centres and an increase in institutional births with skilled attendance are two significant strategies that low- and middle-income countries have used in recent years to overcome the adverse outcomes of maternal and new-born deaths [4]. However, recent data demonstrate the persistent magnitude of the problem and emphasise the importance of developing additional solutions tailored to low- and middle-income countries [5].

Midwifery-led care, an approach which is already widely practiced in developed nations [6]; however, it is a relatively new approach in lower-income countries. In midwifery-led care, a midwife who is well known by their client, provides the care for a low-risk pregnant woman throughout antenatal care, delivery, and the postnatal period, instead of being cared for by various medical staff led by an obstetrician [7]. The primary focus of midwifery-led care is on supporting a healthy physiological pregnancy and labour, and empowering women to give birth naturally with little to no regular intervention [8].

Evidence regarding midwifery-led care links it to a number of advantages, including higher levels of maternal satisfaction and less needless uses of medical interventions [9]. Various studies from high-income countries on the effect of midwifery-led care reported that midwifery-led care could avert about two-thirds of deaths among women and new-borns, reduce obstetric interventions by 13%, and decrease the number of severe adverse maternal outcomes and postpartum incidents [6, 10]. A systematic Cochrane review (2016) of 15 trials involving a total of 17,674 women concluded that midwifery-led care models save infants’ lives, prevent preterm birth, reduce the need for interventions, and improve women’s experiences and clinical outcomes [11]. Additionally, the follow-up Cochrane reviews from 2018 and 2020 also concluded that midwifery-led care prevents stillbirth and preterm birth [12, 13]. Based on this finding, scaling up of midwifery-led care as a paradigm is being advocated by the WHO as well as global health specialists in many nations to enhance maternal and new-born outcomes, lower rates of unnecessary procedures, realise cost savings, and promote natural spontaneous vaginal birth [14–16].

However, there is limited evidence available regarding low- and middle-income countries [17, 18]. Most evidence is from high-income regions [13, 19] and there are currently no pooled estimates on the effectiveness of midwifery-led care to improve maternal and neonatal outcomes in low- and middle-income countries. Pooled effects provide a more comprehensive understanding of the potential effectiveness of midwifery-led care and can inform policymakers and respective stakeholders on the potential implementation of strategies in regions with a high burden of maternal and neonatal deaths [2, 3]. Therefore, this systematic literature review and meta-analysis aimed to assess the effectiveness of midwifery-led care on pregnancy outcomes in low- and middle-income countries.

## Methods

### Search strategies

The study followed the Preferred Reporting Items for Systematic Reviews and Meta-Analyses (PRISMA) guidelines [20] (Additional file 1). This systematic review includes a comprehensive literature search of published scientific articles, in the English language, from January 1, 2000 to July 30, 2022, using the electronic databases which were deemed most relevant for the topics; CINAHL, EMBASE, and PubMed. The search was performed using the following keywords in combination with “AND” and “OR” as specified by the search engine: midwives, midwifery, midwife-led, midwives’ continuum of care, midwifery-led maternity care AND low- and middle-income countries AND pregnancy outcome. The protocol for the review has been registered at the International prospective register of systematic reviews (PROSPERO, CRD 42,022,345,102).

### Inclusion and exclusion criteria

In this systematic review and meta-analysis, we included studies on all pregnant women who received midwifery-led care, including all pregnancy outcomes, maternal and/or neonatal outcomes, in low- and middle-income countries [21], and articles published in English languages between from January 1, 2000 to July, 30 2022. In this systematic review, observational and interventional studies were considered. Studies that did not report on pregnancy outcomes and pregnant women who were cared for during their pregnancy using conventional obstetric care were excluded. Citations without abstract and/or full-text, anonymous reports, editorials, case-reports, case series, and qualitative studies were excluded.

### Study selection and screening

**Fig.1:**
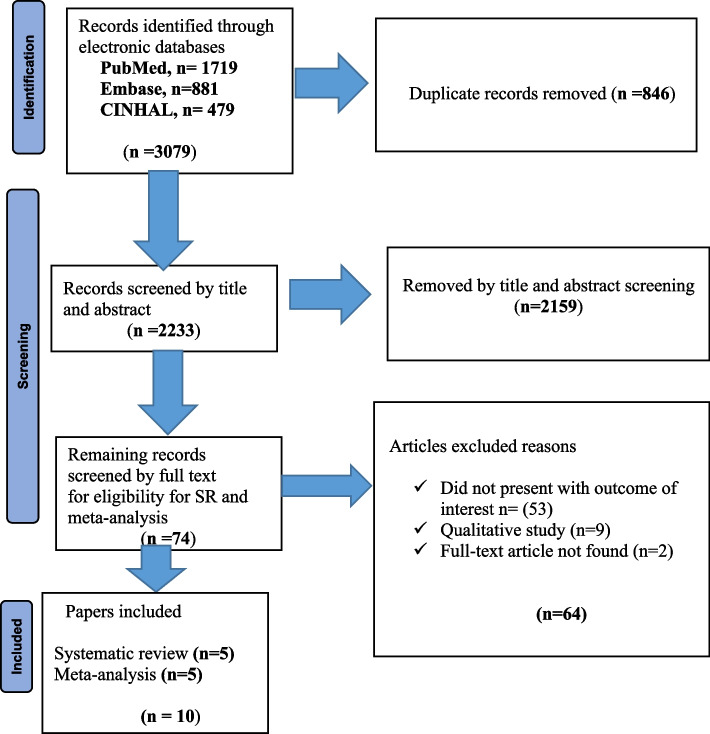
PRISMA flow chart on the effectiveness of midwifery-led care on pregnancy outcomes in low-and middle-income countries, 2022

The retrieved studies were exported to Endnote version 9 http://www.endnote.com/support/ensupport.asp. in order to remove duplicate studies. Before incorporating studies in our systematic review and meta-analysis, we reviewed the title and abstract of each study. The remaining papers were screened for their full-text by two independent reviewers, (RF, WT) using the pre-specified inclusion criteria. Any disagreement was handled based on the specified article selection criteria for the final selection of studies to be included in the systematic review and meta-analysis. The overall study selection process is presented using the PRISMA statement flow diagram (Fig. 1).

### Data extraction

The authors developed a standardised and structured data extraction form in Excel, and the following data were extracted for eligible studies: year of publication, country, setting, study design, intervention recipients, outcomes measured, and the odd ratio effect estimate with a 95% CI. Two authors (RF and WT) independently extracted all-important parameters from each study using the extraction form in collaboration. Then, the extracted data were checked again by two researchers, and disagreements were resolved by tracing back to original articles.

### Data quality

The Joanna Briggs Institute (JBI) critical assessment checklist were used based on the study design, including tool for observational and randomized control trial [22] to rate the quality of the studies that were included. The tool contains information on sample representativeness, article recruitment, sample size data adequacy, detailed study subject and environment descriptions, objective criteria for outcome variable measurement and subpopulation identification, reliability, appropriate statistical analysis, and identification of confounding variables. The quality assessment of all articles was done independently by two researchers.

The quality of the studies assessed using the validated modified version of a quality assessment tool for both observational and randomized studies [23]. The quality assessment tool has nine to eleven questions based on the study design. The score of the quality assessment tool the highest score had the minimum risk of bias. After calculating the overall scores of each study, which are declared low, moderate, and high risk of bias respectively.

After taking the final score of the assessments from the two researchers, using the mean scores, the quality ratings of the included studies were calculated and categorized as high, moderate and low-quality. The quality assessment of the included study was presented in (Supplementary table 1).

### Meta-analysis

A meta-analysis was conducted using STATA 16 software [24] to compute the effects of midwifery-led care on maternal and neonatal outcomes in low- and middle-income countries. We used the random-effects estimator to assess the effectiveness of midwifery-led care on each separate maternal or neonatal outcome. Using the Mantel–Haenszel method [25], typical OR estimates and 95% confidence intervals (CI) were obtained. The random-effects method was used because of the high level of I^2^, which is an important statistic for assessing heterogeneity. I^2^ test statistical findings of 25, 50, and 75% were categorised as having low, moderate, and high heterogeneity, respectively [26]. The Egger regression asymmetry test was used to evaluate the publishing bias [27]. The findings of the included studies were first presented using a narrative synthesis and followed by a meta-analysis chart.

## Results

### Study selection process

The initial search identified 3079 records across three databases, out of which 846 were marked as duplicates. The remaining 2233 records underwent abstract screening. Of those, 2159 did not meet the inclusion criteria. This left 74 full-text articles, which were then assessed for eligibility, and in this step, 64 studies were excluded due to failure to present relevant results, the research being qualitative, or the absence of full-text articles. Finally, ten studies were included for review, of which five were eligible for the meta-analysis and the remaining five were included for systematic review because they were not fit for meta-analysis due to the findings using chi-square and percentages. The PRISMA flow diagram is presented in Fig. 1.

The quality assessment showed that the included studies were meet the evaluation criteria.

### Characteristics of included studies

This review and meta-analysis incorporated studies from low-income countries [28] and middle-income countries [29–37]. Among the included studies, two studies from Iran and Ethiopia were quasi-experimental [28, 29], two studies from China were randomised control studies [31, 32], three studies from China and Palestine were cohort studies [33–35], one study from South Africa was a mixed methods study [30], one study from Nepal was a comparative study [36] and one study from Palestine was a case control study [37]. The included studies were published between 2000 and 2022. Despite all studies meeting the eligibility criteria, the articles varied considerably with regard to the investigated outcomes. In the included studies, all care was provided by midwives, except in one study in which the care was provided by nurse-midwives. The included studies varied regarding risk status of participating women, ranging from low- to high-risk women. The practising settings varied and included clinics, health centres, and hospitals. Midwives in the included studies further varied in experience from junior to expert midwives and duration of training. However, the level of education was not clearly stated in the included studies. The sample of participants in this study ranged from 110 pregnant women [31] to 24,594 participants [30]. The general description of the included studies is presented in (Supplementary table 2).

### Content of midwifery-led care

Midwifery-led care with a caseload team approach was offered by one study [34], a team of midwifery-led care model was offered by seven studies [28, 30–35] and two studies compared midwifery-led care with conventional care [28, 29]. The way in which midwifery-led care was provided differed across studies.

Most of the included studies provided midwifery-led care during the antenatal, delivery, and the postnatal period [28–31, 33–35, 37], and in two studies midwives provided delivery and postnatal care [33, 37].

In most of the included studies, the midwives provide care for pregnant women from Antenatal care to delivery and immediate postnatal care [28, 29, 31–34, 36, 37], and in some of the study the midwives were involved in complications; and management of maternal and new-born infections [30].

### Effectiveness of midwifery-led care on maternal and neonatal outcomes

In the systematic review, the following maternal and neonatal outcomes were used for measuring the effectiveness of midwifery-led care vaginal birth: the modes of birth (Caesarean section vs instrumental birth), episiotomy, birth status (live birth, stillbirth or early neonatal death, preterm birth), the APGAR score at 5 min, birth weight, admission to neonatal intensive care unit, and breastfeeding within one hour. The results of the systematic review indicated that both the maternal and neonatal outcomes during pregnancy, childbirth, and the early postpartum period were significantly improved by midwifery-led care, with no adverse outcomes.

One finding from the systematic review was that midwifery-led care reduced the incidence of birth asphyxia and post-partum haemorrhage < 0.0001[30]. These findings were supported by the evidence that women receiving midwifery-led care showed improved outcomes, with fewer medical interventions [38].

The results from the meta-analysis showed that the rates of emergency Caesarean sections, vaginal births, episiotomies, and neonatal admission time in a neonatal intensive care unit were significantly negatively associated with midwifery-led care. The included studies also showed that the odds of early initiation of exclusive breastfeeding, low birth weight and rate of preterm births were not significantly associated with midwifery-led care [28, 33, 34, 36]. Details regarding the effects on specific outcomes are further discussed below.

### Association between midwifery-led care and emergency Caesarean section

**Fig. 2:**
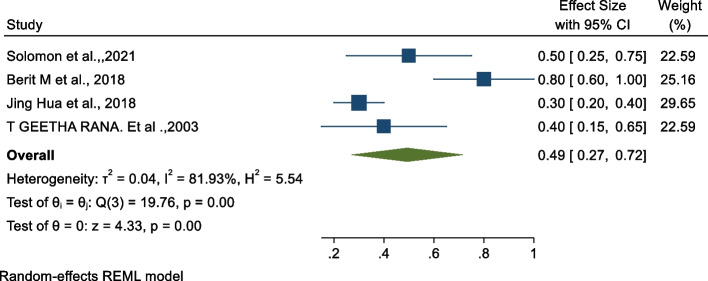
Forest plot of the association between midwifery-led care and emergency Caesarean section

The studies conducted in Iran and China showed that midwifery-led care reduced the risk of emergency Caesarean section compared to those women who did not make use of midwifery-led care [29, 31, 32]. In our meta-analysis, we examined the association between emergency Caesarean section and midwifery-led care [28, 34–36]. The pooled findings revealed that there is a statistically significant negative association between midwifery-led care and emergency Caesarean sections, see Fig. 2 below. The findings revealed that utilising midwifery-led care reduces the odds of emergency Caesarean section by 51% (OR: 0.49, 95% CI: 0.27, 0.72, p < 0.01) as compared to those who did not make use of midwifery-led care. The heterogeneity test indicated I^2^ = 81.93%, hence the random-effects model was assumed in the analysis.

### Association between midwifery-led care and vaginal birth

**Fig. 3:**
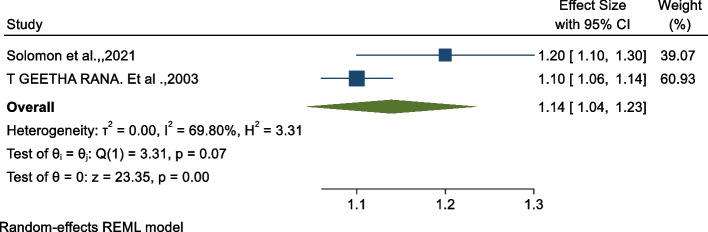
Forest plot of the association between midwifery-led care and vaginal birth

The results showed that midwifery-led care significantly increases the rate of vaginal births [28, 36]. Using the meta-analysis, we examined the association between vaginal birth and midwifery-led care using two studies [32, 34]. As shown on Fig. 3 below, the pooled findings revealed that there is a significant association between midwifery-led care and an increased rate of vaginal births (OR: 1.14, 95% CI: 1.04, 1.23). The heterogeneity test indicated I^2^ = 69.80%, hence the random-effects model was assumed in the analysis.

### Association between midwifery-led care and preterm birth

**Fig. 4:**
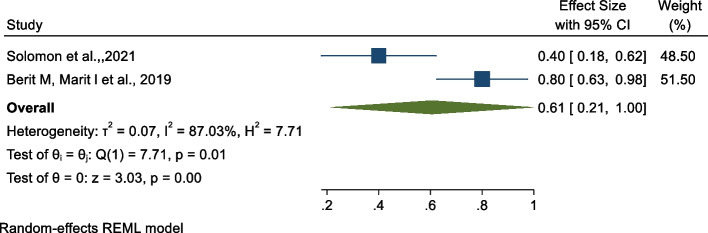
Forest plot of the association between midwifery-led care and preterm birth

The findings of the meta-analysis indicated there is no significant pooled association between midwifery-led care and preterm birth [28, 37]. As shown in Fig. 4 below, there is a borderline statistically non-significant association between midwifery-led care and preterm birth with OR: 0.61, 95% CI: 0.21, 1.00). The heterogeneity test indicated I^2^ = 87.03%, hence the random-effects model was assumed in the analysis.

### Association between midwifery-led care and episiotomy

**Fig. 5:**
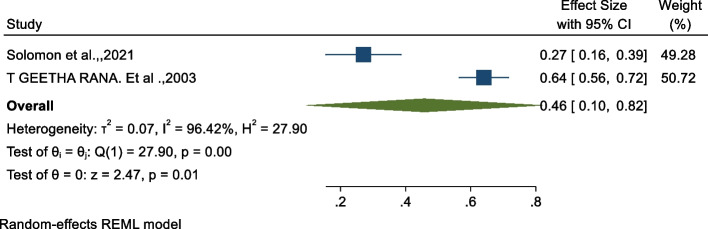
Forest plot of the association between midwifery-led care and episiotomy

We examined the association between episiotomy and midwifery-led care using two studies [28, 36]. As shown in Fig. 5 below, the findings from this analysis revealed that there is a significant association between midwifery-led care and a reduced rate of episiotomies of 54% as compared to those who do not use midwifery-led care (OR: 0.46, 95% CI: 0.10, 0.82, p < 0.01). The heterogeneity test indicated I^2^ = 96.42%, hence the random-effects model was assumed in the analysis.

### Association between midwifery-led care and period of neonatal admission in ICU

**Fig. 6:**
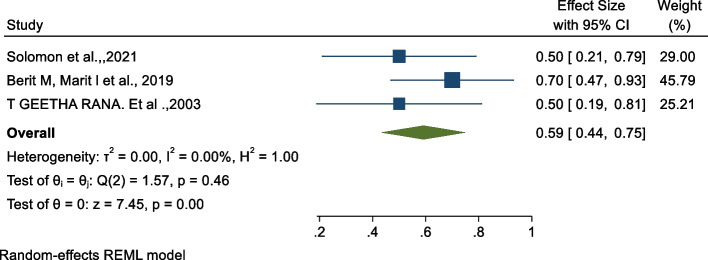
Forest plot of the association between midwifery-led care and period of neonatal admission in ICU

The study from China stated that midwifery-led care reduced the period of admission of neonates in the ICU [29]. The odds ratio of the analysis indicated a negative association between the period of neonatal admission in the ICU and midwifery-led care [28, 34, 35]. As shown in Fig. 6 below, the findings from this analysis revealed that receiving midwifery-led care significantly reduces the odds of a long period of neonatal admission in the ICU by 41% (OR: 0.59, 95% CI: 0.44, 0.75, p < 0.00) as compared to those who did not receive midwifery-led care. The heterogeneity test indicated I^2^ = 0.00%.

### Association between midwifery-led care and early initiations of exclusive breast feeding

**Fig. 7:**
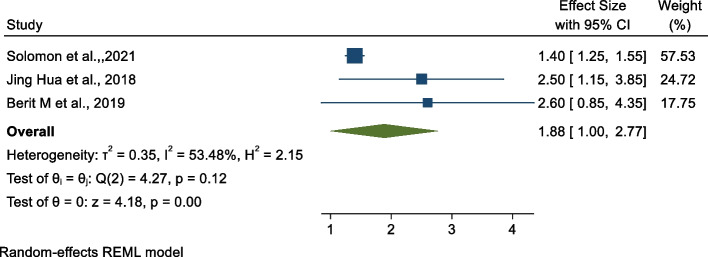
Forest plot of the association between midwifery-led care and early intention of exclusive breast feeding

We examined the association between exclusive breast feeding and midwifery-led care [28, 35, 37]. As shown in Fig. 7 below, the findings from this analysis revealed that there is no significant pooled association between midwifery-led care and early initiation of exclusive breast feeding (OR: 1.88, 95% CI: 1.00, 2.77).

## Discussion

This systematic review and meta-analysis aimed to assess the effectiveness of midwifery-led care to improve maternal and neonatal outcomes in low- and middle-income countries. Over the past 20 years, reducing maternal mortality has been at the top of the global health agenda [39]. It is well known that midwifery-led care can help to improve the quality of care, outcomes, and the efficient use of health care resources by lowering maternal and neonatal mortality and morbidity, lowering stillbirths and preterm births, lowering the number of unnecessary interventions, and raising psychosocial and public health outcomes [40]. However, the current evidence mainly concerns high-income countries [11, 19]. There is little evidence regarding low- and middle-income countries [17, 18], with a lack of pooled estimates of the effectiveness of midwifery-led care on pregnancy outcomes in low- and middle-income countries.

A total of ten studies were eligible for inclusion in this review. According to the quality assessment, nine studies had high methodological quality and one studies had moderate methodological quality.

Findings from the systematic review indicated that midwifery-led care significantly lowered the rate of postpartum haemorrhage and reduced the rate of birth asphyxia. A reduced rate of emergency Caesarean section, increased odds of vaginal birth, a decreased rate of episiotomy and decreased average neonatal admission time in neonatal intensive care unit were significantly associated with midwifery-led care. However, the pooled odds ratio from the meta-analysis shows that early initiation of exclusive breastfeeding and rate of preterm births were not significantly associated with midwifery-led care.

The findings regarding the increased odds of vaginal births with midwifery-led care, [11, 41–43] and the reduced rate of emergency Caesarean sections [11, 41–45] are in line with previous research conducted in high-income countries [41, 42].

A possible explanation for the effectiveness of midwifery-led care in improving maternal and neonatal outcomes could be related to the fact that midwifery-led care focuses on the maintenance of well-being of the women and the promotion of normality by enhancing the physiological capacity of women to give birth with a minimum of – or even no – interventions [46]. The level of knowledge, education, trust, and empowerment of midwives toward midwifery-led care may be connected to the possible impact of the midwives' work during the pregnancy [47]. Women's confidence and comfort during labour, along with the support of the familiar midwife they already know, may have had an impact on the frequency of medical interventions like emergency Caesarean sections [48].

In line with this, the study revealed that midwifery-led care was associated with a reduced rate of episiotomies. Similar findings were reported among women in midwifery-led care among women with a singleton pregnancy, showing that the rate of episiotomy was significantly reduced compared to the rate in women given standard care [11, 41, 43, 46].

Two systematic reviews conducted in high-income countries reported the same conclusion: women who received midwifery-led care were less likely to undergo an episiotomy [11, 49]. Episiotomies are controversial in the majority of developing nations, since they are frequently and sometimes routinely performed, even when not medically indicated [50]. Due to this, many women have considerable health challenges as a result of an episiotomy, often with little to no benefit [51]. When women receive midwifery-led care, midwives are more familiar with their patients, so that information about a delivery plan and the possible interventions are discussed, and closer attention is paid to a woman's individual needs [52].

In this review and meta-analysis, neonatal admission time of neonates in a neonatal intensive care unit was significantly reduced by midwifery-led care, in line with studies from high-income countries [41, 42]. This suggests that midwifery-led care leads to shorter hospital stays, and fewer tests and interventions and the development of a trusting relationship between midwives and expectant mothers may have lowered labour-related stress, which may have in turn decreased the reasons for neonatal admission [38, 52].

The present study concluded that early initiation of exclusive breastfeeding was borderline significant. The possible reason might be that the difference in quality of the included studies affect the pooled effect. Preterm birth was not significantly associated with midwifery-led care. A possible reason might be due to the limited number of studies included in the case of preterm births in the current review. By contrast, studies conducted in high-income countries revealed that midwifery-led care improves the outcomes [11, 53, 54].

### Strengths and limitations

The current study has several strengths. To our knowledge, this study is the first systematic review and meta-analysis evaluating the effectiveness of midwifery-led care to improve pregnancy outcomes in low- and middle-income countries. The meta-analysis provided additional strong evidence to the systematic review. The researchers used extensive and comprehensive search strategies based on a pre-specified protocol. The literature search was systematic and assessed by two independent reviewers within the desired scope. We also adhered to PRISMA guidelines and conducted the quality assessment of the included studies. However, only studies in the English language were included, which could have led to missed research written in local languages. The number of included studies was limited, reflecting the lack of research in this area. As a result, the findings might not be representative of the entire region, meaning that low-income countries, especially eastern Africa, lack adequate studies.

In addition, we were not able to show combined pooled estimates for all outcome variables associated with midwifery-led care because the included studies classified the variables in different ways.

### Implications for practice and research

The implementation of midwife-led care should be taken into consideration as a choice in maternal health care in low- and middle-income countries. We should scale up such interventions as they are critical for providing quality of care during the antenatal, delivery, and postpartum periods. A comprehensive approach should be implemented to prevent adverse pregnancy outcomes including facility-based midwifery-led care. This requires that all responsible bodies, including ministries of health, the respective regional health bureaus, and other stakeholders should work together to reduce maternal and neonatal mortality. Furthermore, additional research is needed on the effects of midwifery-led care on a broader range of outcomes, including longer term follow-up of infants’ development.

## Conclusions

This systematic review and meta-analysis revealed that midwifery-led care has many positive effects on improving several key maternal and neonatal outcomes, including fewer emergency Caesarean sections, higher rates of vaginal births, lower rates of episiotomies, and shorter neonatal stays in intensive care units. Implementing midwifery-led care helps to sustainably enhance maternal and new-born health outcomes, while also empowering midwives to provide better maternal health care.

## Supplementary Information

**Additional file 1. **

**Additional file 2: Supplementary table 1.** Summary of quality assessments using JBI appraisal checklist, 2020.

**Additional file 3: Supplementary table 2.** Characteristic of the included study on the effectiveness of midwifery-led care on pregnancy outcomes, 2022.

## Data Availability

All relevant materials and data supporting the findings of this review are included within the manuscript.

## Abbreviations

CI: Confidence interval
PPH: Postpartum haemorrhage
MLC: Midwifery-led care
OR: Odds ratio
PRISMA: Preferred reporting items for systematic review and meta-analysis
