# Supplementary material for: Effectiveness of midwifery-led care on pregnancy outcomes in low- and middle-income countries: a systematic review and meta-analysis
Source: BMC Pregnancy Childbirth. 2023 May 26;23:386. doi: 10.1186/s12884-023-05664-9 (PMC10214693; doi:10.1186/s12884-023-05664-9)
Supplement: Supplementary file 2 — Additional file 2: Supplementary table 1. Summary of quality assessments using JBI appraisal checklist, 2020. [file 12884_2023_5664_MOESM2_ESM.docx]

Supplementary table 1. Summary of quality assessments using JBI appraisal checklist, 2020.

| **Authors** | **Items (Q) on Joanna Briggs Institute instrument^a^** | **Raw score %** | **Overall Risk assessment** |
| --- | --- | --- | --- |
|  | Q1 Q2 Q3 Q4 Q5 Q6 Q7 Q8 Q9 Q10 Q11 |  |  |
| Azam et al., 2021[29] | 1 1 1 1 1 1 0 1 1 | 8/9=88.8% | Low |
| Oosthuizen et al., 2019[30] | 1 1 1 1 1 1 0 1 1 0 1 | 9/11=81.8% | Low |
| Chunyi et al., 2013[31] | 1 1 1 1 1 1 1 1 1 | 9/9=100% | Low |
| Xiu et al., 2018[32] | 1 1 1 1 1 1 1 0 1 | 8/9=88.8% | Low |
| Ngai et al., 2010[33] | 1 1 1 0 1 1 1 1 1 1 1 | 10/11=90.9% | Low |
| Solomon et al., 2021[34] | 1 1 1 1 1 1 1 1 1 | 9/9=100 | Low |
| Berit et al., 2019[35] | 1 1 1 1 0 1 1 1 1 1 1 | 10/11=90.9% | Low |
| Jing et al., 2018[36] | 1 1 1 1 1 1 1 1 1 0 1 | 10/11=90.9% | Low |
| T GEETHA. et al., 2003 [37] | 1 0 1 1 0 1 U 1 1 | 6/9=66.6% | Moderate |
| Berit & Lien et al., 2019[38] | 1 1 1 1 0 1 0 1 1 1 | 8/10=80% | Low |

**^a^** Yes=1, No= 0, U= Unclear and Unapplicable
