# Supplementary material for: Effectiveness of midwifery-led care on pregnancy outcomes in low- and middle-income countries: a systematic review and meta-analysis
Source: BMC Pregnancy Childbirth. 2023 May 26;23:386. doi: 10.1186/s12884-023-05664-9 (PMC10214693; doi:10.1186/s12884-023-05664-9)
Supplement: Supplementary file 3 — Additional file 3: Supplementary table 2. Characteristic of the included study on the effectiveness of midwifery-led care on pregnancy outcomes, 2022. [file 12884_2023_5664_MOESM3_ESM.docx]

Supplementary table 2**:** Characteristic of the included study on the effectiveness of midwifery-led care on pregnancy outcomes, 2022.

| **No** | **Authors/ Year** | **Study design** | **Sample size** | **Setting** | **Sampling methods** | **Participants** | **Intervention description** | **Outcomes measured** |
| --- | --- | --- | --- | --- | --- | --- | --- | --- |
| 1 | Azam et al., 2021[29] | Quasi-experimental | N= 200 pregnant women  MLC= 100  SC=100 | **Type:** Health centre  **Location**: Kashan, Iran | Non-probability sampling | Pregnant women | Midwives follow the selected mothers using the designed continuous care model through a team of midwives, and mothers access care every time by responsible midwives. | Mode of delivery (Caesarean section, vaginal birth), Early initiations of lactation. |
| 2 | Oosthuizen et al., 2019[30] | Longitudinal | N= 24,594 | **Type:** Health districts  **Location:**  Tshwane, South Africa | Purposive sampling | Pregnant women | ‘CLEVER’ intervention package in the five intervention units using observational methods and then implementations were carried out following high-priority areas: essential childbirth and new-born care including labour monitoring; complications; and management of maternal and new-born infections. | Reduced fresh stillbirths, meconium aspiration and birth asphyxia. |
| 3 | Chunyi et al., 2013[31] | Randomized controlled trial | N= 110 Pregnant women  MLC=55  CG=55 | **Type:** Hospital  **Location**: Fudan University, Shanghai, China | Random sampling | Pregnant women | The midwifery care was provided by a group of midwives. The midwives were in charge of antenatal care procedures for women who had been allocated to the intervention group. The midwife usually focused on antenatal check-ups, consultation, making birth plans, parent education, and collaborated with obstetricians and other health professionals as necessary. Each woman had the chance of having continuous one-to-one care from the onset of labour to 2 h post-partum. | Mode of delivery (Caesarean section, vaginal birth), amount of vaginal bleeding, neonatal APGAR score, women’s psychological state, and satisfaction with care |
| 4 | Xiu et al., 2018[32] | \| Randomized  controlled trial \| \| --- \| | N= 666 Pregnant women.  MLC= 333  CG= 333 | **Type**: Hospital  **Location**:   \| Fujian Province, \| \| --- \|   China | \| Random  sampling \| \| --- \| \|  \| | Pregnant women | The women received a midwife‐led pathway model of care with birth companion by a midwife and a family member during the whole process of delivery. | Mode of delivery (vaginal birth, Caesarean section) , duration of labour, post-partum haemorrhage, 5‐minute APGAR scores, rates of intra-partum and post-partum maternal complication, rates of neonatal asphyxia, and neonatal hospitalisations |
| 5 | Ngai et al., 2010[33] | Retrospective cohort | N= 226 pregnant women | **Type**: Hospital  **Location**: China | Stratified random sampling | Pregnant women | The MNBU features midwife-led care, a birth plan, complementary therapies and ‘two-to-one’ care by a midwife and a birth companion of the woman’s choice. | Mode of delivery (Caesarean section, vaginal birth) APGAR score. |

| **No** | **Author /Year** | **Study design** | **Sample size** | **Setting** | **Sample method** | **Participants** | **Intervention description** | **Outcomes measured** |
| --- | --- | --- | --- | --- | --- | --- | --- | --- |
| 6 | Solomon et al., 2021[34] | Quasi- experimental | N= 1178 pregnant women  MLC=589  SC=589 | **Type:** Hospital  **Location**: North Shoa, Ethiopia | Stratified cluster sampling techniques | Pregnant women | Pregnant women who had received their entire antenatal, labour and birth and immediate postnatal care from one of the participating midwives. Care during labour and birth was provided in the labour ward at the intervention hospitals in 8 h shifts. If the care of the woman in labour took longer than 8 h, the midwife handed over responsibility to another midwife in the team. | Mode of delivery (vaginal birth, Caesarean section, instrumental) preterm birth, episiotomy, perinatal trauma, induction, and augmentation of labour live birth, still birth or early neonatal death. APGAR score at 5 min, birth weight, admission to neonatal intensive care unit (NICU), and breastfeeding within one hour |
| 7 | Berit et al., 2019[35] | Retrospective cohort | N= 2201 | **Type**: Hospital  **Location**: Nablus, Palestine | Purposive sampling | Pregnant women | The midwives followed up their case-load of women by individual consultations throughout pregnancy. They assessed health status and risk factors and referred to appropriate specialist care when necessary and the midwife continued to follow up women with detected health risks in collaboration with specialist care. | Women satisfaction and exclusive breast feeding. |
| 8 | Jing et al., 2018[36] | Cohort | N= 1730 | **Type:** Clinic  Location: China | Convenience | Pregnant women | A small group of midwives provide care to the pregnant women, including antenatal check-ups, consultation, birth planning, parenting education. They are also responsible for providing care during labour, birth, and the immediate post-partum period, | Maternal wellbeing, delivery mode (Caesarean vs vaginal birth), early initiations of exclusive breastfeeding. |
| 9 | T GEETHA. et al., 2003 [37] | Comparative | N= 988 pregnant women | **Type:** Hospital  Location: Patan, Nepal | Convenience | Pregnant women | The CMU handles both high- and low-risk deliveries. Admission is conducted by a duty midwife, who judges risk status on the basis of history, examination, and review of antenatal chart | Artificial rupture of membrane, augmentation, episiotomy. |
| 10 | Berit & Lien et al., 2019[38] | Observational case-control | N=186 pregnant women | **Type:** Hospital  Location: west bank, Palestine | Convenience | Pregnant women | Midwives travelled to rural villages scattered in different regions of the West Bank, that offered the midwife-led continuity model care. | Mode of delivery, induction of labour, maternal anaemia, blood transfusion, preterm, birth weight, APGAR score, neonatal death, new-born admitted to ICU. |

**N=** Number of participants **MLC=** Midwifery-led care **C=** Controlled group **SC=** Standard group **ICU**= Intensive care unit **MNBU**=Midwives-led birth unit
